# An Evolutionary Perspective on Vector-Borne Diseases

**DOI:** 10.3389/fgene.2019.01266

**Published:** 2019-12-17

**Authors:** Jeffrey R. Powell

**Affiliations:** Yale University, New Haven, CT, United States

**Keywords:** vector-borne diseases, evolution, genetics, mosquitoes, pathogens

## Abstract

Several aspects of the biology of the three players in a vector-borne disease that affect their evolutionary interactions are outlined. A model of the origin of a human–human cycle of vector-borne diseases is presented emphasizing the narrowing of the niche experienced by the pathogen and vector. Variation in the expected rates of evolution of the three players is discussed with the rapid rate of pathogen evolution providing them with advantages. Population sizes and fluctuations also affect the three players in very different ways. The time since the origin of a vector-borne disease likely determines how stable the interactions are and thus how easily the disease might be eliminated. Stability and variation are also linked. Human technological advances are rapidly upsetting the previously relatively slow coevolutionary adjustment of the three players. Finally, it is pointed out that development of quantitative coevolutionary models specifically addressing details of vector-borne diseases is needed to identify parameters most likely to break transmission cycles and thus control or eliminate diseases.

## Introduction

Vector-borne diseases represent a three-species interaction problem. The interconnectedness of the three players is the outcome of coevolutionary processes acting, in some cases, over long periods of time and, in other cases, very short periods of time. To fully understand their dynamics and evolution, the vector, pathogen, and vertebrate host need to be considered simultaneously. Here I discuss the kinds of factors that an evolutionary biologist would focus on when trying to understand how the coevolutionary process has molded the present state of the interactions. By doing so, one can better understand ongoing dynamics and anticipate future changes.

I also note at the outset that I focus on mosquito-borne diseases when presenting examples, although most of the issues and principles are general to vector-borne diseases, at least those transmitted by arthropods.

## General model of origins

**Figure 1:**
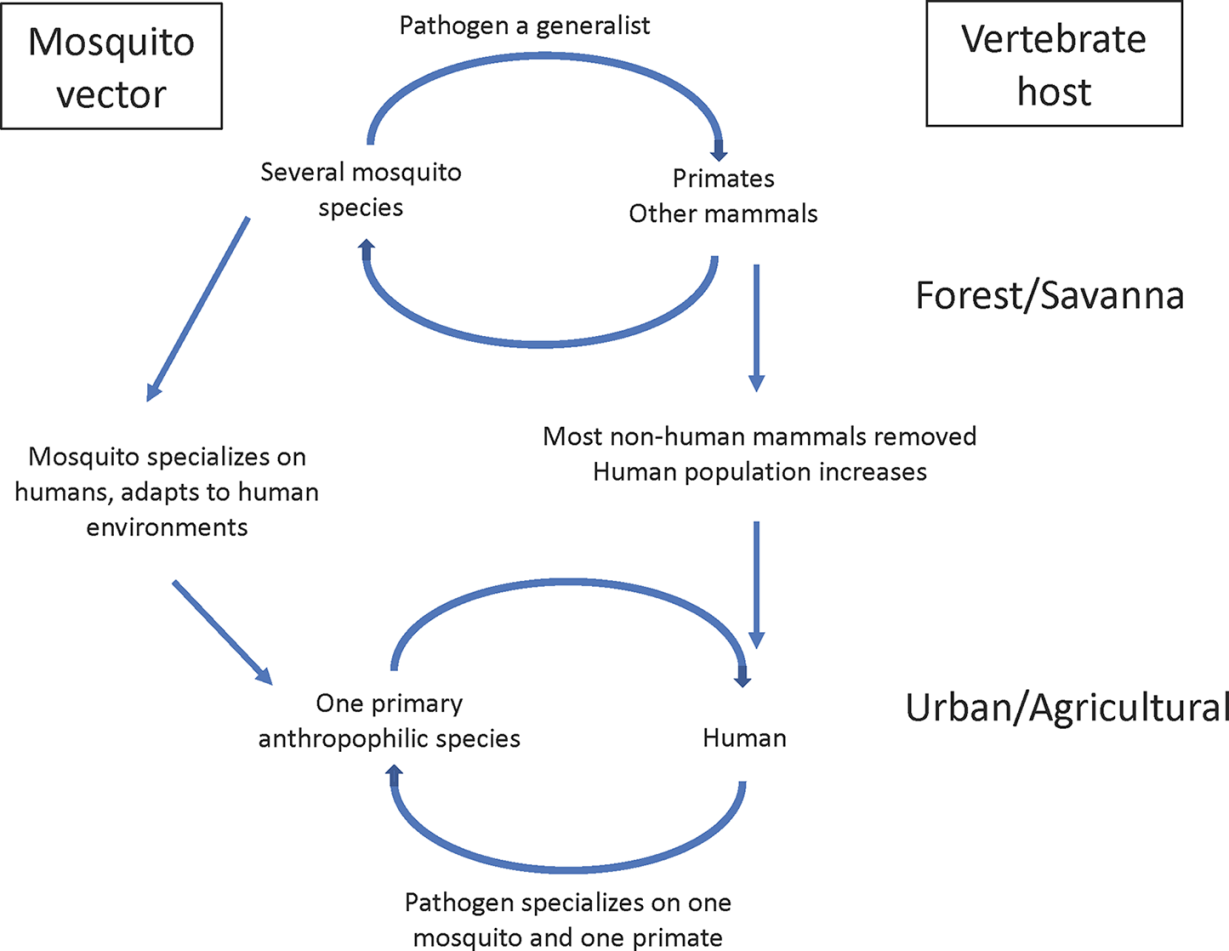
Schematic model of origin of human-human vector-borne disease.

**Figure 1** illustrates a general model of the origin of many mosquito-borne diseases. Before emerging as human diseases, in many well-studied cases the ancestral three players in a natural ecosystem have been identified. For mosquito-borne diseases, multiple species of mosquitoes and multiple vertebrates were, and many still are, involved in the sylvatic transmission cycle. Thus, pathogens were selected to be generalists in both the mosquito and vertebrate host stages. Once a pathogen becomes a human disease, it experiences a major change, a shrinking of host range, often to one vertebrate host (humans) and one primary mosquito species. Selection for specialization begins. Using viruses as an example, Halbach et al. (2017) and Warren and Sawyer (2019) reviewed the issue of virus adaptation to humans and point out that only a small minority of animal viruses (less than 0.1%) are capable of replicating in human cells. Selection is severe for which of the many sylvan pathogens can establish in humans.

It is not a coincidence that the most important mosquito vectors of human diseases are closely associated with human habitats; indeed they are not simply “associated with” but clearly adapted to be intimately integrated into the human ecosystem. It is plausible that most mosquito-borne diseases had their origins *after* an appropriate mosquito adapted to human habitats. One or a few humans acquired a future human disease-causing pathogen due to “bridge vectors,” vectors that were primarily non-human biters but would occasionally bite a human if they encroached on their territory. When the infected human(s) rveturned to their communities, a human–human cycle could only be established if a competent vector was already present in sufficient density in the human habitat. In this scenario, the mosquito led the way, the pathogen followed. Just as only a small minority of animal pathogens can infect humans, only as a small minority of the estimated 3,500 species of mosquitoes live in human habitats.

The importance of mosquito adaptation to human habitats is illustrated by three of the most notorious mosquito vectors from three different genera. It is likely not a coincidence that all three have their ancestral home in sub-Saharan Africa, the ancestral home of man.

*Anopheles gambiae* s.l. in sub-Saharan Africa is a complex of species that breed in many niches, some natural, but many human-constructed such as sunny pools left by cattle or in rice fields (Touré et al., 1998b; Coetzee et al., 2000; della Torre et al., 2002). The species/populations of this complex of mosquitoes breeding closest to humans transmit most human malaria in sub-Saharan Africa. *Aedes aegypti* is also native to sub-Saharan Africa where ancestral natural populations exist today that breed in tree holes and adults take blood meals from non-human sources (Gouck, 1972; Lounibos, 1981; McBride et al., 2014). As human villages began to form and expand in Africa, *Ae. aegypti* was the one member of its genus in Africa to begin breeding in stored water in villages especially in West Africa that experiences prolonged dry seasons (Powell et al., 2018). It also evolved a preference for humans as a blood source. It was this domesticated form of *Ae. aegypti* (subspecies *Ae. aegypti aegypti*) that spread around the tropical and subtropical world due to human movements. *Culex pipiens* s.l. is also likely a native African species, ancestrally a bird feeder like most Culex (Harbach, 2012). Outside Africa, populations of this species are largely human-associated including in uniquely urban habitats like sewers and septic tanks. Its common name, the house mosquito, describes the close association of adults with the indoors and its preference for human blood meals.

One curious observation in considering these three mosquito vectors is that two of them, *An. gambiae* s.l. and *C. pipiens* s.l., are complexes of cryptic species, subspecies, and forms of various genetic and taxonomic status. [Harbach (2012) suggested using the term Pipiens Assemblage rather than “complex” for *C. pipiens* s.l.] These have likely arisen in most cases as a consequence of evolving associations with humans, very recent from an evolutionary standpoint. While *Ae. aegypti* has two named subspecies that have considerable morphological variation (McClelland, 1974), there is little evidence of the sort of taxonomic complexity associated with reproductive isolation observed in the other two groups (although see Dickson et al., 2016). Poorly studied populations of *Ae. aegypti* on Indian Ocean islands off the East Coast of Africa may harbor new taxa of some rank (unpublished observations), although this remains to be clarified.

**Figure 1** is an oversimplification because most human diseases are not transmitted by a single mosquito species nor necessarily confined to a single vertebrate host. In the case of the human malaria parasite, *Plasmodium falciparum*, while members of the *An. gambiae* complex have been the primary vectors, even in sub-Saharan Africa other vectors exist, most prominently *An. funestus.* In the case of yellow fever, it is probably true that for centuries it was transmitted to humans by a single species, *Ae. aegypti*, although today *Ae. albopictus* must be considered a potential vector since it is competent to transmit the yellow fever virus (YFV) and lives in close association with humans (Lourenco de Oliveria et al., 2003). Also, in South America there is a well-documented non-human primate (monkey) reservoir for yellow fever maintained by *Haemagogus* mosquitoes (Bates and Roca-Garcia, 1945). Despite these caveats, it still seems safe to conclude that, in general, the number of vectors and vertebrate hosts shrink when a pathogen emerges as a human disease.

## Three perspectives

In **Tables 1**–**3**, I present the evolutionary perspective of the three different players involved in vector-borne diseases. These are attributes of two players that would maximize the fitness of the third. Here I purposely use teleological language as it is a simple and straight-forward way to communicate sometimes complex concepts. This should not be misconstrued to mean there is any intention on the part of the three players, simply that natural selection *appears* to be intentional when it acts to maximize the fitness of organisms through a non-intentional process. I do not presume these tables to be exhaustive; rather they are presented as a useful framework to think about the problem.

**Table 1:** Example of factors to consider in the evolution of vector-borne disease from the standpoint of the pathogen.

What the pathogen wants:
From the vertebrate host:
1. High density
2. Susceptible
3. Attractive to mosquito
4. Infective for a long time, but not fatal
5. Behave to be available to the mosquito
6. Poor immune response to pathogen
From the mosquito:
1. Frequent blood meals
2. High competence to transmit, rapid transit from gut to saliva
3. Innate immune system that does not inhibit pathogen growth
3. Longevity

**Table 2:** Example of factors to consider in the evolution of vector-borne disease from the standpoint of the mosquito vector.

What the mosquito wants:
From the pathogen
1. Low pathogenicity to mosquito, not affect fitness
2. If it affects mosquito fitness, rapid passage through mosquito
From the vertebrate host (human)
1. High density of blood sources
2. Behave to be available to mosquito
3. Blood with sufficient nutrients to produce as many eggs as possible
4. Not harbor other pathogens that reduces mosquito fitness
5. Not develop technologies to lower mosquito fitness

**Table 3:** Example of factors to consider in the evolution of vector-borne disease from the standpoint of the vertebrate/human host.

What the vertebrate host (human) wants:
From the pathogen
1. Low pathogenicity
2. Short infections
3. Short infectious period
4. Easily recognized antigens for immune response and vaccine development
From the mosquito
1. Low density
2. Poor host seeking
3. Low competence to transmit
4. Short life time

**Table 1** presents a listing of traits of mosquitoes and humans that would optimize the spread and survival (fitness) of the pathogen. From the pathogen’s perspective, the ideal mosquito is one that frequently takes blood meals from both infected and uninfected humans and provides ideal physiology for the pathogen to quickly traverse from the midgut to saliva. The mosquito must also live long enough to take at least one additional blood meal after an initial infection. The ideal vertebrate host would be in high density, attractive to the relevant mosquito species, behave to be available for blood meals (e.g., not use window screens, insect repellents, etc.), be susceptible to infection, not be able to mount an effective immune response, and achieve an infective pathogen titer in its blood for as long as possible. However, it is not to the advantage of the pathogen that the vertebrate host dies quickly and is removed from the ecosystem; the longer it remains alive and infective to mosquitoes, the better for the pathogen. Ewald (1994) discusses these issues in more detail.

Point 3 in second part of **Table 1** has received considerable attention and is covered in detail in other contributions to this issue. Suffice it to say the interaction of pathogens with the vector’s innate immune system is complex and intense. In the case of viruses, many insect-specific viruses are known and mosquito genomes often harbor large numbers of viral sequences (e.g., Houe et al., 2019; Pischedda et al., 2019). This has likely increased the rate of evolution of the major antiviral immune response, RNA silencing (Bernhardt et al., 2012). Oson and Blair (2015) and Kramer and Ciota (2015) discuss and review arbovirus–mosquito interactions in much more detail with a focus on the mosquito innate immune system.

**Table 2** is a list of traits of the pathogen and vertebrate host (humans) ideal for a mosquito vector. Primarily, the mosquito is best served if the pathogen has no negative effect on female mosquito fitness. As far as known, because only females transmit pathogen, males are not affected by the pathogen. Similar to the pathogen perspective, lethality to the vertebrate host is undesirable as it removes a sources of blood meals. Also, as with the pathogen, high density of the preferred vertebrate for blood meals, behaving to be available, is desirable. The vertebrate host blood should have sufficient nutrients to support development of many eggs. If the vertebrate host blood has other pathogens or molecules (e.g., antibodies), they are minimally detrimental to the mosquito. The vertebrate host (i.e., humans) should not develop technologies to limit mosquito fitness, or only technologies that minimally affect mosquito fitness (e.g., window screens) or are relatively easy to overcome (e.g., insecticides).

In this regard, work on the effect of Plasmodium infection on anopheline mosquito vectors is instructive. For some decades, studies addressing the question of whether there is a fitness cost for a female mosquito to transmit malaria produced confusing and conflicting results. In some studies, infected mosquitoes showed no lowered fitness compared to unaffected siblings, while in other studies, a decrease in fitness was demonstrated. Ferguson and Read (2002) cut through the confusion in an analysis of 22 published studies. They recognized that there were a variety of species of Plasmodium and several species of Anopheles mosquitoes used in the studies. They subdivided the studies into ones where the combination of mosquito species and plasmodium species is known to occur in nature and studies where the combination of mosquito and parasite was not known to be natural. There were 10 studies in each category with two where the natural vector was not known. In every case where the combination was known to occur in nature, there was no detectable fitness effect on mosquitoes, whereas in 7/10 unnatural combinations, there was a detectable fitness cost to the infected mosquito. This clearly shows that over the time that the natural association existed, selection had co-adapted the particular Plasmodium species and mosquito species such that infections had no detectable harm on the mosquito, a situation beneficial to both the vector and pathogen. More detailed information on the interactions between malaria parasites and their mosquito hosts is reviewed in Lefevre et al. (2018).

From a physiological and biochemical standpoint, one might expect multicellular eukaryote pathogens like malaria to have a cost of infection for mosquitoes; it is less obvious that viral pathogens should "cost" much for a mosquito, i.e., they require fewer resources from the host to reproduce. On the other hand, viruses lyse their host cells upon completion of their life cycle. Few studies have been performed on the fitness cost to mosquitoes to be infected with a human disease-causing virus. Lambrechts and Scott (2009) reviewed studies and found only 12; none were performed on the four major arboviruses (YFV, DENV, CHIKV, ZIKV) affecting humans using the normal route of infection (blood meal). More recently, Padilha et al. (2018) found that *Ae. aegypti* infected with the ZIKV had decreased locomotor activity but had no effect on more directly related fitness traits, viability, and egg production. Vogels et al. (2017), contrary to expectations, found that *C. pipiens* infected with West Nile virus decreased their host (bird) seeking behavior. It is safe to conclude that we know very little about the cost of arboviral infection to mosquito hosts for the most important human arbovirus diseases.

**Table 3** presents the traits of the pathogen and vector most important to the vertebrate host. These are quite obvious and largely involve minimal harm to the vertebrate for a minimal time. Small populations of mosquitoes are desirable with short life spans. It is best for the vertebrate if the pathogen has simple, easily recognized antigens, such that the human could mount an immune response, acquire immunity, and develop an effective vaccine.

In comparing **Tables 1**–**3**, it is clear that the three players have both overlapping and conflicting interests in certain aspects of the system. Overall, what is good for the mosquito in obtaining multiple blood meals is good for the pathogen (higher production of eggs for mosquito, higher transmission rate for pathogen), while negative for humans. The human immune system aims to avoid or shorten infection, while the pathogen’s goal is to prolong infection so it is transmitted to a new host. Longevity of the mosquito is beneficial for the mosquito as well as the pathogen; the more blood meals taken, the greater egg production (mosquito fitness) and the higher number of vertebrate hosts infected (pathogen fitness). As long as the pathogen does not affect mosquito fitness, length of infection in the mosquito is irrelevant for the mosquito.

## Rates of evolution

**Figure 2:**
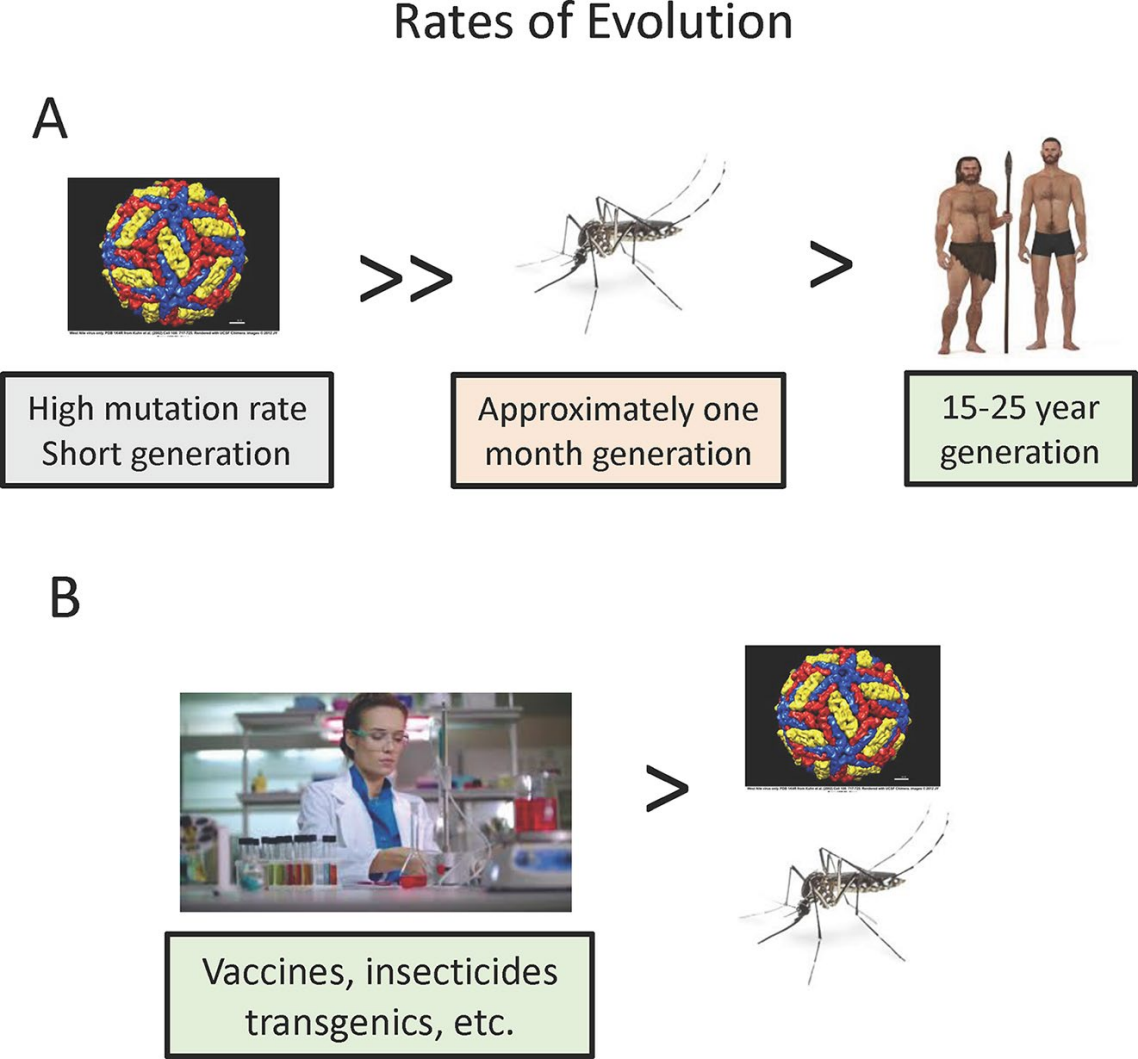
Relative rates of evolution of pathogens, vectors, and vertebrate hosts. **(A)** Natural situation. **(B)** Effect of human culture and technology.

The three players in vector-borne diseases differ greatly in their potential for evolutionary change and thus the rate at which we might expect them to adjust to the complex three species interactions (**Figure 2A**). Whether the vector-borne pathogens are prokaryotes or eukaryotes, they undergo many more generations per unit of time, e.g. transmission cycles, than do the vector or human host, and thus can evolve more rapidly over absolute time. While mutation rates in some eukaryote pathogens may not vary much from vectors or mammals, RNA viruses are the most important viral vector-borne pathogens (yellow fever, dengue, etc.), and these are known to have particularly high mutation rates, at least one hundred times higher than the typical eukaryotes. Coupled with their short generation time, this makes arboviruses particularly fast in evolutionary potential. This rapid rate of evolution of pathogenic RNA viruses has been observed twice recently in regard to efficiency of spread by mosquitoes. A single amino acid substitution has been implicated in the increased efficiency of chikungunya transmission in *Aedes albopictus* (Tsetsarkin and Weaver, 2011) and three nucleotide changes in the West Nile Virus increases the efficiency of transmission by *C. pipiens* and *Culex tarsalis* (Moudy et al., 2007; but see Anderson et al., 2012 for conflicting results). These changes occurred in a time frame of a few years rather than the millennia usually considered in evolutionary biology. Weaver (2006) discusses in more detail the dynamics of arbovirus evolution.

While rates of biological evolution (changes in genomes) depend on mutation rates and generation time, humans have upset this simple relationship through rapid cultural changes (**Figure 2B**). Of most relevance to vector-borne disease evolution, technological advances such as development of insecticides, vaccines, and potentially transgenic vectors are most important in affecting the interactions of the three players. These changes can occur more rapidly than any biological evolution.

Any analysis of the “arms race” among the three players needs to take into consideration these fundamental differences among the players in the rate at which they can evolve.

## Mode of reproduction

Related to potential rates of evolution is the mode of reproduction, in particular whether the reproductive cycles involve recombination. Recombination generates more diversity and thus greater evolutionary flexibility, more different phenotypes for selection to act on. Most vectors and vertebrate host are diploid, sexually reproducing organisms, so recombination is a given. However, recombination rates in vectors can vary considerably. As one example, two of the most important mosquito vectors, vary four-fold in recombination rate per physical distance: 1.3 cM/Mb for *Anopheles Coluzzi* (Pombi et al., 2006) and 0.33 cM/Mb for *Ae. aegypti* (Matthews et al., 2018).

Some, but not all, vector-borne human pathogens have recombination. Eukaryotes like Plasmodium and Filaria have a well-known sexual stage that results in recombination (Conway et al., 1999; Small et al., 2016; Wong et al., 2018). Recombination in arboviruses is perhaps less well known and appreciated. Within Flaviviridae, recombination is known to occur in the DENV, Japanese encephalitis, and St. Louis encephalitis but surprisingly not in YFV or West Nile (Twiddy and Holmes, 2003). In the family Togoviridae, another virus of considerable medical importance, CHIKV, also has been documented to recombine (Filomatori et al., 2019).

## Time

Closely related to rates of evolution is the time the evolutionary process has had to adjust the three players to one another. This varies from disease to disease. As one example of old associations, the most important disease-causing arboviruses had their origin in Africa where a plethora of viruses are transmitted by members of the genus *Aedes* to various African primates. A single African *Aedes, Ae. aegypti*, evolved to breed in close contact with human settlements and began to prefer humans for blood meals. This form of *Ae. aegypti* associated with human habitats has spread throughout the tropical and subtropical world. When African-native viruses such as YFV, DENV, ZIKV, and CHIKV also left Africa, it should not be a surprise that *Ae. aegypti* is the major vector to humans due to their previous long association in Africa (Powell, 2018). *P. falciparum* transmitted by *A. gambiae* s.l. to humans is very likely an old association dating to the Neolithic period in Africa, ∼12,000 years ago (Coluzzi, 1999).

These long associations can be contrasted to West Nile virus in the US. This virus was first detected in the US in 1999. As noted above, the virus evolved over ∼10 years to be adapted to be efficiently transmitted by a North American-native species of mosquito, *Culex tarsalis*, and to have effectively eliminated previous genotypes of the virus in North America (Moudy et al., 2007). It is curious that this virus’ very rapid ability to adapt to new vectors is not reflected in its adaptation to a new vertebrate host. While West Nile virus is generally spread among birds by *Culex* mosquitoes, these mosquitoes occasionally infect a human where the virus eventually dies, i.e., humans are a dead-end host never having a high enough titer to infect another mosquito. It is unclear why a pathogen "should" infect a dead-end host unless there is no selective pressure to be successfully transmitted by a second host if the primary host is abundant enough. The dead-end host is simply a random “mistake.” Furthermore, for most pathogens that have multiple vertebrate hosts, the hosts are closely related (e.g., primates) and present a more homogeneous environment for the pathogen than would humans and birds.

## Variation and stability

When a pathogen is transmitted by one species of vector and infects one vertebrate host (**Figure 1A**), there is selective pressure on the pathogen to specialize, become highly efficient at replicating in both the vector and vertebrate. Eventually, with sufficient time the pathogen should evolve to be able to replicate and be transmitted by every individual vector and vertebrate host. In turn, vector and vertebrate host will “fight” the pathogen, evolve mechanisms to reduce any harmful effects caused by the pathogen infections. But given greater evolutionary rates inherent in most pathogens relative to the other two players, the pathogen has the advantage. Eventually, at equilibrium, we expect all three players to arrive at a stable state with some intermediate level compromise among the varying interests of the different players (**Tables 1**–**3**). One expectation of a stable state is there is little variation within each of the players with regard to how they are interacting. This expectation is not met in most vector-borne diseases.

In the case of vertebrate hosts, the classic example is malaria and various human hemoglobin variants that make their carriers resistant to Plasmodium infection or not to have severe consequences of infection (Piel et al., 2010). Genetic variation in humans for susceptibility to arbovirus infections is less well studied. Two studies, both on dengue, have revealed human genetic variants that affect dengue infections (Coffey et al., 2009; Khor et al., 2011).

Similarly, different strains of pathogens bearing the same name (e.g., *P. falciparum,* dengue virus, etc.) vary in their ability to infect both arthropods and vertebrate hosts. Given their often very large population sizes and high mutation rates, it is not surprising that vector-borne pathogens harbor considerable genetic variation that can affect their behavior in both the vector and vertebrate host.

More surprising is the great deal of variation among individual vectors (best studied for arboviruses and mosquitoes) in their ability to transmit a disease with which they have shared a long evolutionary history. Souza-Neto et al. (2019) reviewed 91 publications in which *Ae. aegypti* originating from many localities were studied for their ability to be infected and in some cases transmit (infection in saliva) many different arboviruses including the most important they are known to transmit in nature (YFV, DENV, CHIKV, and ZIKV). The take-away message is that considerable variation exists among individuals and populations in their susceptibility to infection/transmission. While variation in competency phenotypes among mosquitoes may be due to random or environmental variation, at least some of the variation has a genetic basis. For example, **Table 4** shows results of tests for vector competence for three viruses using the same virus isolate, studied in the same lab using identical procedures. Strains of this mosquito coming from different localities vary considerably in their susceptibility to be infected with these viruses. Similar studies have been performed for malaria with similar results (e.g. Lambrechts et al., 2005; Lambrechts and Scott, 2009).

**Table 4:** Examples of vector competence studies on Aedes aegypti for three of the major viruses this species transmits, Zika, dengue, and yellow fever.

Origin of mosquito strain	Virus	Infection rate	Reference
Salvador, Brazil	Zika DAK AR	100%	Roundy et al., 2017
Rio Grande, Texas	“	40%	
Singapore	Dengue Guinea C	90%	Sim et al., 2013
Bangkok	“	10%	
Guatemala	Yellow Fever Asibi	2%	Tabachnick et al., 1985
Kwa Dzivo Kenya	“	57%	

The geographic origin of mosquitoes tested is in first column with the virus in the second. Infection rate (third column) is the percent of females that blood fed on infective blood that became infected. Note that in this table are presented studies using the same strain of virus and assayed in the same laboratory using identical methods on both strains of mosquitoes assayed.

As noted earlier, this is something of an enigma: why haven’t pathogens that are transmitted by one (or very few) mosquitoes not evolved to more efficiently infect all females in vector populations? Especially viruses with high mutation rates, large population sizes, and short generation time, have an advantage in this arms race. A possible answer to this enigma is to question the relevance of laboratory studies of vector competence to the natural environment with actual epidemiological consequences, an issue also raised by Randolph and Nuttall (1994). For example, Miller et al. (1989) document an epidemic of urban yellow fever in Nigeria in which only 7% of the local *Ae. aegypti* population was capable of transmitting the YFV in laboratory studies. Either the rate of transmission inferred from laboratory studies is an artifact, or this low rate of competence to transmit is sufficient to maintain an outbreak of yellow fever. This raises the question of how much of the variance in phenotype (in this case ability to transmit the YFV) is due to genetic variation and how much to environmental variation (e.g., laboratory environments). That at least part (much)? of the phenotypic variation has a genetic basis has been demonstrated multiple times by, for example, selecting for mosquito strains that differ in vector competence (e.g. Wallis et al., 1985; Collins et al., 1986; Miller and Mitchell, 1991). And the differences observed in data in **Table 4** must have a large genetic component when the environment and tested pathogen are held constant.

Regardless of possible artifacts in these laboratory studies, there is little doubt that vectors harbor an unexpectedly high degree of heterogeneity in ability to transmit, unexpected because, over time, fast evolving pathogens should have adapted better to their vectors. The good news is this may imply relative instability of vector-pathogen associations such that breaking the cycle is easier than if they were more stable. Similarly, it is potentially possible to use this genetic variation in vectors to affect genetic modification without recourse to controversial transgenic methods (Powell and Tabachnick, 2014; Xia et al., 2019).

## Population sizes and fluctuations

While all three players in these systems may experience population fluctuations, they do so to widely differing degrees. Humans, by and large, have relatively stable population sizes at least over the period of years or decades. Mosquitoes have moderate fluctuation in numbers, especially in temperate zones or localities with pronounced dry-wet cycles.

**Figure 3:**
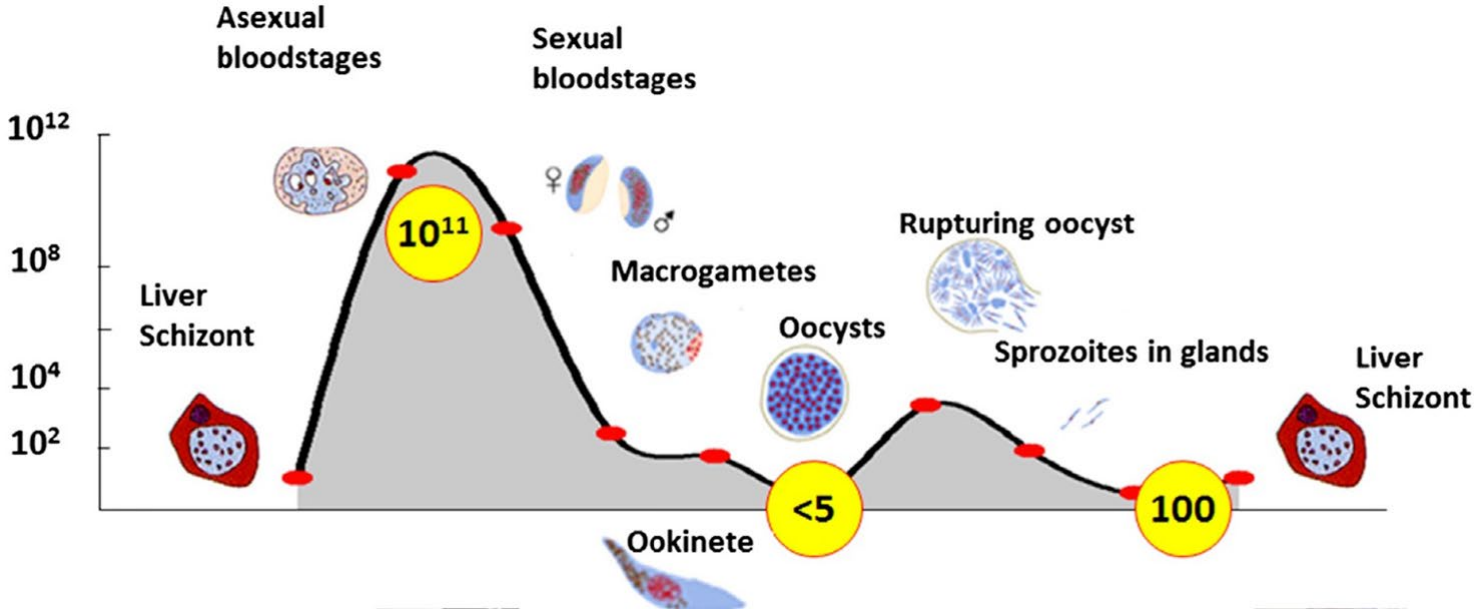
Population dynamics of a plasmodium infection. Yellow circles are estimated numbers at each stage. From Sindon (2017) with permission.

When turning to the third player, the pathogen, the pattern is radically different in both population size and fluctuations. As one example, Plasmodium parasites may fluctuate in a single transmission cycle from 10^11^ to five or even less (**Figure 3**). By comparison, the total human population today is about 8 X 10^9^, less than 1/10 the size of a Plasmodium infection in a single host! Similar large fluctuations in population size occur for arboviruses during a single transmission cycle (Grubaugh and Ebel, 2016; Grubaugh et al., 2016; Lambrechts and Lequime, 2016). Interestingly, the part of the transmission cycle with the highest population size differs among diseases. In malaria, clearly the largest Plasmodium populations are in humans (vertebrate host) (**Figure 3**) whereas in West Nile virus, the largest populations and genetic diversity are found in mosquitoes (Jerzak et al., 2007).

In terms of absolute population sizes of vectors, two contrasting situations have been identified. In the case of *Ae. aegypti*, it is surprising that often humans may out-number the local mosquito vector. For example, the effective population (N_e_) size of *Ae. aegypti* has been shown to be 50–700 and absolute census size 1,000–5,000 (Sheppard et al., 1969; Lounibos, 2003; Carvalho et al., 2015; Endersby et al., 2011; Saarman et al., 2017). These small mosquito populations almost certainly reflect a meta-population structure due to very limited dispersal, but this is the level that is most important for evolutionary change to occur.

The situation is quite different when considering Anopheline vectors of malaria in Africa. Effective population sizes of *A. gambiae* (Lehmann et al., 1998) and *An. arabiensis* (Taylor et al., 1993) have been measured to be ∼2,000–7,000. The census size of *An. gambiae* in a village in Mali with ∼700 human inhabitants was estimated to be ∼10–30,000 (Toure et al., 1998a). Anopheline population sizes larger than the human population is also reflected in annual human biting rates. In localities in Uganda this can range up to 18,000 per human with up to 50 *An. gambiae* found in a single hut (Kilama et al., 2014). Given that a female mosquito takes 1–3 blood meals in a lifetime on average, this means mosquito population sizes must be much larger than the human population size.

This extreme cycle of population growth and crashes experienced by most vector-borne pathogens has important evolutionary consequences. During times of large population due to very rapid growth (flush), selection is relaxed and large numbers of new mutations are generated some of which may even have lowered fitness (Grubaugh and Ebel, 2016). On the other hand, rapid increase in population size may lead to increased fitness. In the context of S. Wright’s adaptive landscape model (Wright, 1932; Arnold et al., 2001) this is a time when populations can explore a greater amount of the genotype space and potentially reach higher adaptive peaks requiring passage through lowered fitness space.

## Human activities

In attempting to stem mosquito-borne diseases, humans have developed a large number of tactics that affect both mosquitoes and pathogens making humans the fastest evolving member of the tripartite dance (**Figure 2B**). Obvious measures such as vaccines and drugs directly affect pathogens. The use of screens on windows and doors lowers the density of available blood meals. Removal of larval breeding sites reduces mosquito populations.

The use by humans of insecticides and its potential effects on a mosquito vector competence provides an example of the close coupling of the three players, sometimes in unexpected ways. In three cases either exposure to an insecticide or insecticide resistant mosquitoes have been shown to have increased competence to transmit a pathogen: Alout et al. (2013) for Anopheles and malaria, Knecht et al. (2018) for *Aedes albopictus* and ZIKV, and Atyme et al. (2019) for *C. pipiens* and West Nile virus. Thus a human action meant to control one player (vector) has an effect on the third (pathogen).

Analogous to insecticides, drugs used to treat diseases put evolutionary pressure on pathogens to evolve resistance. This has occurred multiple times for malaria and can occur fairly rapidly from an evolutionary perspective (Haldar et al., 2018). There are a few studies that show drug resistant pathogens may differ in their behavior in vectors compared to non-resistant strains (Suchet et al., 1977; Delang et al., 2018) or that patients treated with a drug (chloroquine for malaria) may increase infectivity for mosquitoes (increased production of oocysts; Wilkinson et al., 1976). This illustrates the need to consider the three-player interactions even if a control method is designed to target only a single player.

And when (if)? the employment of genetically modified mosquitoes that reduce or eliminate the ability of mosquitoes to support pathogen transmission is successful, selection on pathogens to bypass the blocks will be very strong.

## Modeling

In attempting to understand the evolution of vector-borne diseases, quantitative modeling can be very insightful especially in identifying parameters of greatest importance. Unfortunately, little attention has been paid to this subject. Modeling of vector-borne diseases has focused on issues like disease prevalence and rates of transmission rather than on the evolutionary biology of species interactions. The now classic work of May and Anderson (1983) and Ewald (1994) broke ground on ecological and evolutionary approaches to infectious diseases. Galvani (2003) more recently reviews studies that take an evolutionary perspective. But in all of this work, explicit attention to *vector-borne* infectious diseases receives only passing attention. A major problem is that dealing with a three-species interaction system is more complex than the usual two-species models. Attempts at modeling three-species systems have provided some insights (Nuismer and Doebeli, 2004), but again the models explored are hard to apply to vector-borne diseases. Holingsworth et al. (2015) discuss in more detail challenges of modeling vector-borne diseases.

As already noted, labeling vector-borne diseases a three species interaction problem is in many cases an oversimplification. Often, a pathogen may infect multiple vertebrate and vector hosts, and vectors and vertebrates may be simultaneously infected with multiple pathogens. Issues like cross-immunity by vertebrates and vectors arise; i.e., a vertebrate or vector may become resistant to one pathogen that also affects another pathogen. Added to this is the potential effect of microbiota on vectors and their efficiency to transmit pathogens. It has been shown that microbial infections of mosquitoes can change vector fitness and competence in significant ways (Ramirez et al., 2012; Dennison et al., 2014; Dickson et al., 2017; Saraiva et al., 2018). How the vertebrate host microbiota may affect infections of vector-borne pathogens seems has not been examined. Thus, the full panoply of distinct species involved quickly becomes very large further complicating attempts at modeling.

Despite these complexities and caveats, development of quantitative models specifically focused on the biology of species interactions in vector-borne diseases can lend insights into important issues even if simplifying assumptions are necessary. As one example, stability of any given vector-borne disease can be important in designing control measures. Coluzzi (1999) makes a convincing case that malaria around the Mediterranean basin was relatively easy to eradicate using DDT because the vectors and climate were marginal for malaria transmission making this situation unstable in contrast to sub-Saharan Africa where the three species interactions are very stable. The variation that exists among the players in most vector-borne diseases implies many of these systems are not particularly stable.

One purpose of the above considerations is to suggest the kinds of issues any models need to consider. Each of the three players have their own population size and fluctuations, rates of evolution, etc. And each disease has it own age since the transmission cycle arose. While quantitative models with equations and computer simulations are highly useful, it is also true that scientists construct models in their minds when they consider a complex system and design experiments to learn about the system. It is hoped that the foregoing discussion might help clarify and refine the models we all carry in our heads.

## Author Contributions

The author confirms being the sole contributor of this work and has approved it for publication.

## Conflict of Interest

The author declares that the research was conducted in the absence of any commercial or financial relationships that could be construed as a potential conflict of interest. 

The handling editor declared a past co-authorship with the author JP.
